# Filling space with hypercubes of two sizes – The pythagorean tiling in higher dimensions

**DOI:** 10.1112/mtk.12152

**Published:** 2022-06-07

**Authors:** Jakob Führer

**Affiliations:** ^1^ Institute of Analysis and Number Theory Technical University of Graz Kopernikusgasse 24/II Austria

## Abstract

We construct a unilateral lattice tiling of $R^{n}$ into hypercubes of two differnet side lengths *p* or *q*. This generalizes the Pythagorean tiling in $R^{2}$. We also show that this tiling is unique up to symmetries, which proves a variation of a conjecture by Bölcskei from 2001. For positive integers *p* and *q*, this tiling also provides a tiling of ${\left(Z/\left(p^{n}+q^{n}\right)Z\right)}^{n}$.

## 1 INTRODUCTION

In 1907, Minkowski [18] conjectured that any tiling of $R^{n}$ into *n*‐dimensional unit hypercubes whose center points form a lattice has to contain two hypercubes that share a full facet. Keller [10] generalized this conjecture by allowing any tiling of $R^{n}$ into *n*‐dimensional unit hypercubes. Perron [19] proved Keller's conjecture for n⩽6 in 1940 and Hajos [8] proved Minkowski's original conjecture in 1942.

In 1992, Lagarias and Shor [12] proved Keller's conjecture to be false, in particular they showed it is false for n⩾10. The gaps were filled by Mackey [14] for dimensions 8 and 9 and Brakensiek et. al. [4] for dimension 7, showing that 7 is the smallest dimension where Keller's conjecture is true. For additional information, see the survey on unit cubes by Zong [24].

In $R^{2}$, there are some related results.

**FIGURE 1:**
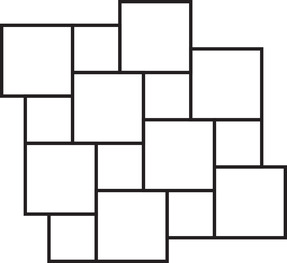
The Pythagorean tiling

- The well‐known Pythagorean tiling (Figure 1) is a lattice tiling of $R^{2}$ into squares of two sizes as well as the unique unilateral and equitransitive tiling into squares of two sizes [7, 15].
- There are exactly eight different unilateral and equitransitive tilings of $R^{2}$ into squares of three sizes [2, 7, 15, 20].
- $R^{2}$ can be tiled into squares of pairwise distinct integer side lengths in $2^{{ℵ}_{0}}$ ways. For n⩾3, this is not possible not even if we only require neighboring cubes to have different sizes [5].
- Sprague [22] gave the first example of a tiling of a square into squares of pairwise different integer side lengths (see also [6] and [7]).
- Meir and Moser [17] asked whether a square of area $\sum_{n=1}^{\infty }1/n^{2}=\pi^{2}/6$ can be tiled by the squares of side length 1/n for $n\in Z_{⩾1}$. The problem remains open but Januszewski and Zielonka [9] showed that for 1/2<t⩽2/3, the square of area $\sum_{n=1}^{\infty }1/n^{2t}$ can be tiled by the squares of side length $1/n^{t}$ for $n\in Z_{⩾1}$ and Tao [23] showed that for every 2/3<t<1 there exists an $n_{0}\in N$ such that the square of area $\sum_{n=n_{0}}^{\infty }1/n^{2t}$ can be tiled by the squares of side length $1/n^{t}$ for $n\in Z_{⩾n_{0}}$.

In order to state the main result, we need the following definitions: A *tiling* is a partition into closed sets without holes, called tiles, whose pairwise intersection is of Lebesgue measure zero. We further call sets of Lebesgue measure zero *null sets*. For tilings of $R^{n}$ into hypercubes, we call a tiling *unilateral* if no two hypercubes of the same size share a full facet and *equitransitive* if any two hypercubes of the same size can be mapped to each other by an isomorphism that keeps the tiling unchanged. *Lattice tilings* are tilings where the center points of all hypercubes of the same size form a lattice and are therefore also equitransitive.

**FIGURE 2:**
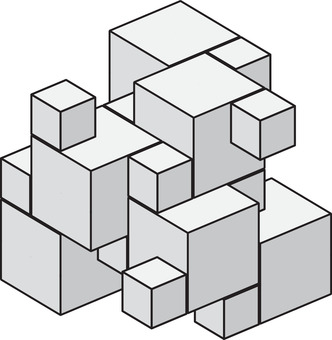
Roger's filling

In 2001, Bölcskei [3] proved that Roger's filling (Figure 2) is the only unilateral and equitransitive tiling of $R^{3}$ into cubes of two sizes and conjectured this to be true for any higher dimension.

Conjecture 1 (Bölcskei [3]) In every dimension *d*, there exists precisely one equitransitive unilateral tiling by *d*‐dimensional cubes of two sizes.

We prove the following variation of the conjecture, where we consider lattice tiling instead of equitransitive tilings:

Theorem 1 Let $p,q\in R_{+}$ with q<p and $n\in N_{⩾2}$. There exists exactly one unilateral lattice tiling of $R^{n}$ into hypercubes of side length *p* or *q* up to symmetries.

Remark 1 Since lattice tilings are equitransitive, in regards to existence Theorem 1 is stronger than Conjecture 1 and in regards to uniqueness it is the other way around.

We write [a,b] for {a,a+1,a+2,…,b−1,b}⊆Z and $e_{i}$ for the *i*th standard unit vector in $R^{n}$.

## 2 EXISTENCE

We split Theorem 1 into two parts: The first is Theorem 2 and the other is dealt with in § 4.

Theorem 2 Let $p,q\in R_{+}$ with q<p and $n\in N_{⩾2}$. There exists a lattice tiling of $R^{n}$ into hypercubes of side length *p* or *q*.

Remark 2 After a preprint of this article appeared on arXiv, the author was informed by Mihalis Kolountzakis that Theorem 2 follows from [11, Theorem 8].

Let *A* be the following n×n matrix:


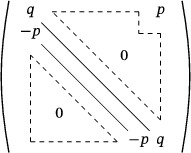




Theorem 2 follows directly from the following Lemma.

Lemma 1 ${\left[0,q\right]}^{n}\cup {\left[0,p\right]}^{n-1}\times \left[q,q+p\right]$ is a system of representatives of $R^{n}/AZ^{n}$ up to null sets.

We split the proof of Lemma 1 into four claims.

Claim 1 Every element in $R^{n}$ has a representative in ${\left[0,q\right)}^{n-1}\times R$.

Proof of Claim 1 We can inductively add multiples of the *k*th column of *A* to find a representative of any element in ${\left[0,q\right)}^{k}\times R^{n-k}$ up to k=n−1.□

Claim 2 Every element $x={\left(x_{1},x_{2},\text{…},x_{n}\right)}^{T}\in {\left[0,q\right)}^{n-1}\times R_{⩾0}$ with $x_{n}⩾p+q$ has a representative in ${\left[0,q\right)}^{n-1}\times \left[0,x_{n}-q\right]$.

Proof of Claim 2 Consider the following matrix *A*_1_:


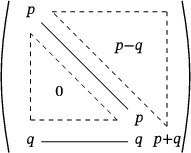

Let $a_{1},..,a_{n}$ be the columns of *A* and $b_{1},\text{…},b_{n}$ the columns of *A*_1_. We obtain *A*_1_ in the following way.

- $b_{1}=a_{n}$.
- $b_{k}=b_{k-1}-a_{k-1}$, for k∈[2,n].


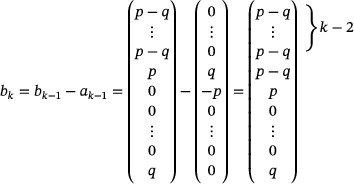

Now let l∈[1,n] such that $x_{i}<p$ for all i<l and $x_{l}⩾p$, then $x-b_{l}$ is a representative of *x* in ${\left[0,q\right)}^{n-1}\times \left[0,x_{n}-q\right]$.□

Claim 3 Every element $x={\left(x_{1},x_{2},\text{…},x_{n}\right)}^{T}\in {\left[0,q\right)}^{n-1}\times R_{<0}$ has a representative in ${\left[0,q\right)}^{n-1}\times \left[x_{n}+q,p+q\right)$.

Proof of Claim 3 Let $b_{1},\text{…}b_{n}$ be as before and let l∈[1,n] be such that $x_{i}⩾q-p$ for all i<l and $x_{l}<q-p$. Then $x+b_{l}$ is a representative of *x* in ${\left[0,q\right)}^{n-1}\times \left[x_{n}+q,p+q\right)$.□

By iteratively applying the last two claims, we get a representative in ${\left[0,q\right)}^{n-1}\times \left[0,p+q\right)$ for every element.

Now let $x\in \left({\left[0,q\right)}^{n-1}\times \left[0,p+q\right)\right)\setminus \left({\left[0,q\right)}^{n}\cup {\left[0,p\right)}^{n-1}\times \left[q,q+p\right)\right)$, and again let l∈[1,n] such that $x_{i}<p$ for all i<l and $x_{l}⩾p$. From the definition of *x*, it follows that l<n and $x-b_{l}$ is a representative of *x* in ${\left[0,q\right)}^{n}$ because $x_{n}>q$.

Claim 4 The following holds: $\det\left(A\right)=p^{n}+q^{n}$.

Proof of Claim 4 We calculate the determinant with the Laplace expansion along the first row.

$$\det\left(A\right)=q\left|\begin{array}{cccc} q & \; & \; & \;\\ -p & \;\ddots & \; & \;\\ & \;\ddots & \;\ddots & \;\\ & \; & \;-p & \;q \end{array}\right|+{\left(-1\right)}^{n-1}p\left|\begin{array}{cccc} -p & \;q & \; & \;\\ & \;\ddots & \;\ddots & \;\\ & \; & \;\ddots & \;q\\ & \; & \; & \;-p \end{array}\right|=q^{n}+p^{n}$$

□

We now prove Lemma 1. The parallelepiped *P* spanned by the columns of *A* is a system of representatives (up to null sets) of $R^{n}/AZ^{n}$ with Lebesgue measure $\det\left(A\right)=p^{n}+q^{n}$. Let $P={⊎}_{i\in N}P_{i}$ be a decomposition of *P* into sets of points that share the same translation into $C:={\left[0,q\right)}^{n}\cup {\left[0,p\right)}^{n-1}\times \left[q,q+p\right)$ by the previous reductions and let $Q_{i}$ be the corresponding translated sets. Every set $P_{i}$ and therefore also $Q_{i}$ is measurable since it is constructed by intersections of halfspaces. Since *P* is a system of representatives (up to null sets), the sets $Q_{i}$ have pairwise intersection of measure 0 and therefore

$$p^{n}+q^{n}=\lambda \left(P\right)=\sum_{i\in N}\lambda \left(P_{i}\right)=\sum_{i\in N}\lambda \left(Q_{i}\right)⩽\lambda \left(C\right)=p^{n}+q^{n}.$$

Consequently, ${\left[0,q\right]}^{n}\cup {\left[0,p\right]}^{n-1}\times \left[q,q+p\right]=C={⋃}_{i\in N}Q_{i}$ up to null sets, which concludes the proof.□

Remark 3 Two different points in the interior of ${\left[0,q\right]}^{n}\cup {\left[0,p\right]}^{n-1}\times \left[q,q+p\right]$ cannot be representatives of the same element in $R^{n}/AZ^{n}$ as this would imply that there were neighborhoods of the two points corresponding to the same set in $R^{n}/AZ^{n}$ of non‐zero Lebesgue measure.

To show that the tiling is unilateral, the followimg Lemma is sufficient.

Lemma 2 Let i∈[1,n] and let $e_{i}$ be the *i*th standard unit vector, then $pe_{i}\notin AZ^{n}$ and $qe_{i}\notin AZ^{n}$.

There are two points in the interior of ${\left[0,q\right]}^{n}$ with difference $pe_{i}$ and therefore they are both different representatives of $R^{n}/AZ^{n}$, so $pe_{i}\notin AZ^{n}$. Analogously, there are two points in the interior of ${\left[0,q\right)}^{n}\cup {\left[0,p\right)}^{n-1}\times \left[q,q+p\right)$ with difference $qe_{n}\notin AZ^{n}$. Suppose $qe_{i}\in AZ^{n}$ for i<n, then also $qe_{i}-a_{i}=pe_{i+1}\in AZ^{n}$, a contradiction to the previous statement.□

## 3 SYMMETRIES AND PERIODICITY

The group of symmetries of the tiling has to keep the cube invariant, so it is a supgroup of the hyperoctahedral group $B_{n}$ which has order $2^{n}n!$. Again we work with the matrix formulation of the problem and write the group operations as matrix multiplications from the left. Here the hyperoctahedral group can be written in the following way: $B_{n}≅B_{n}^{\prime }$ where

$$B_{n}^{\prime }:=\left\{S\in {\left\{0,\pm 1\right\}}^{n\times n}|\text{every}\;\text{row}\;\text{and}\;\text{column}\;\text{contains}\;\text{exactly}\;\text{one}\;\pm 1\;\text{entry}\right\}$$

and we find the biggest supgroup of $B_{n}^{\prime }$ which fixes $AZ^{n}$. In other words, $S\in B_{n}^{\prime }$ is in the stabilizer of the tiling ⇔(SA)X=A has an integer solution

$$\Leftrightarrow A^{-1}S^{-1}A\in Z^{n\times n}.$$

Theorem 3 $S:={\left(s_{i,j}\right)}_{i,j\in [1,n]}\in B_{n}^{\prime }$ is in the stabilizer of the tiling ⇔ *S* is of the following form.

- $s_{i,j}=s_{i+1,j+1}$ for i<n,j<n.
- $s_{i,n}=-s_{i+1,1}$ and $s_{n,j}=-s_{1,j+1}$ for i<n,j<n.
- $s_{n,n}=s_{1,1}$.


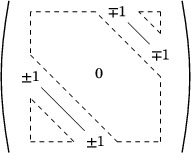

‘⇐’: Let *S* be as above and *i* such that $s_{i,1}=\pm 1$. Without loss of generality, $s_{i,1}=1$. Now

$$Sa_{j}=\left\{\begin{array}{cc} qe_{i+j+1}-pe_{i+j}=a_{i+j-1} & j⩽n-i\\ qe_{n}+pe_{1}=a_{n} & j=n-i+1\\ -qe_{-n+i+j-1}+pe_{-n+i+j}=-a_{-n+i+j-1} & j⩾n-i+2. \end{array}\right.$$

‘⇒’: Let $v:=qe_{i}\pm pe_{j}$ such that $v\neq a_{i}$ and i≠j. Then

$$a_{i}-v=\left\{\begin{array}{cc} -pe_{i+1}\mp pe_{j} & i⩽n-1\wedge j\neq i+1\\ pe_{1}\mp pe_{j} & i=n\wedge j\neq 1\\ -2pe_{j} & i⩽n-1\wedge j=i+1\\ 2pe_{1} & i=n\wedge j=1. \end{array}\right.$$

In the first two cases, $a_{i}-v$ is not in $AZ^{n}$ because it is a vector connecting two inner points of ${\left[0,q\right)}^{n}$. Since $a_{j}-2pe_{j}$ is also connecting two inner points of ${\left[0,q\right)}^{n}$, $a_{i}-v\notin AZ^{n}$ and therefore $v\notin AZ^{n}$. Since every vector $Sa_{j}$ is of the form $qe_{k}\pm pe_{l}$ or the negation of it for all j∈[1,n] there exists i∈[1,n] such that $Sa_{j}=\pm a_{i}$. Now suppose $s_{i,j}=\pm 1$, then $Sa_{j}=\pm a_{i}$ and therefore

$$s_{i+1,j+1}=\left\{\begin{array}{cc} \pm 1 & i⩽n-1\wedge j⩽n-1\\ \mp 1 & i=n\wedge j⩽n-1\\ \mp 1 & i⩽n-1\wedge j=n\\ \pm 1 & i=n\wedge j=n. \end{array}\right.$$

□

On the periodicity of the tiling along the coordinate axis, we have the following result:

Theorem 4 Let p,q∈N with gcd(p,q)=1. Then $p^{n}+q^{n}=\min\left\{l\in N|le_{i}\in AZ^{n}\right\}$ for all i∈[1,n]. In particular, there exists a unilateral lattice tiling of ${\left(Z/\left(p^{n}+q^{n}\right)Z\right)}^{n}$ into hypercubes of side length *p* or *q*.

Let i∈[1,n]. From Cramer's rule, we know that the solution of $Ax=e_{i}$ in $Q^{n}$ is of the form $x=k/\det\left(A\right)=k/\left(p^{n}+q^{n}\right)$ for a $k={\left(k_{1},\text{…},k_{n}\right)}^{T}\in Z^{n}$ and therefore $\left(p^{n}+q^{n}\right)e_{i}\in AZ^{n}$. More specifically, $k_{i}=\det\left(A_{i}\right)/\left(p^{n}+q^{n}\right)$, where $A_{i}=\left(a_{1},\text{…},a_{i-1},e_{i},a_{i+1},\text{…},a_{n}\right)$. Calculating the determinant with the Laplace expansion along the last column, we get $\det\left(A_{i}\right)=q^{n-1}$ and since $\gcd(p^{n}+q^{n},q^{n-1})=1$ the statement follows.

Remark 4 For q=2 and p=1, the tiling gives a lower bound on the maximal number of hypercubes of side length two that can be packed in ${\left(Z/\left(2^{n}+1\right)Z\right)}^{n}$, which coincides with the optimal solution (see [1]). Finding such packings is used to determine the Shannon capacity of odd cycles (see [13, 16 21]).□

## 4 UNIQUENESS

We now show that every unilateral lattice tiling of $R^{n}$ into hypercubes of two sizes is equivalent to the tiling described in the previous sections. It is known that such a tiling does not exist if we only use cubes of one size (see Hajós [8]) and so we can describe the tiling by means of two cubes of different size, translated by $BZ^{n}$ for an n×n matrix *B* of full rank.

Lemma 3 Two small cubes cannot touch.

Assume the contrary. Then there exists $x:={\left(x_{1},\text{…},x_{n}\right)}^{T}\in BZ^{n}$ with $|x_{i}|⩽p$ for all i∈[1,n]. Now *x* connects two inner points of the big cube, a contradiction.□

Lemma 4 A small and a big cube that properly touch share a corner.

Let *T* be a big cube and *S* a small cube that properly touch. Without loss of generality, let $T={\left[0,q\right]}^{n}$ and *S* properly touches *T* in $\left\{q\right\}\times R^{n-1}$. We write $S=\left[q,q+p\right]\times {⨂}_{j=2}^{n}\left[x_{i},x_{i}+p\right]$ where $x_{i}\in \left(-p,p+q\right)$ for all i∈[2,n].

**FIGURE 3:**
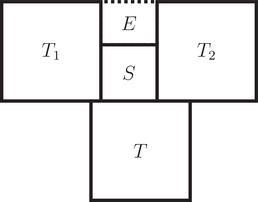
The 2‐dimensional setup of the fist case in the proof of Lemma 4

Assume *S* touches *T* in a full side but *S* and *T* do not share a corner, then $x_{i}\in \left[0,q-p\right]$ for all i∈[2,n] and without loss of generality $x_{n}\notin \left\{0,p-q\right\}$. There have to be big cubes $T_{1},T_{2}$ that touch *T* in $\left\{q\right\}\times {⨂}_{j=2}^{n-1}\left[x_{i},x_{i}+\varepsilon \right]\times \left[x_{n}-\varepsilon ,x_{n}\right]$ and $\left\{q\right\}\times {⨂}_{j=2}^{n-1}\left[x_{i},x_{i}+\varepsilon \right]\times \left[x_{n}+q,x_{n}+q+\varepsilon \right]$, respectively, for a ε>0. Therefore, $T_{1}=\left[q,2q\right]\times {⨂}_{j=2}^{n-1}\left[y_{i},y_{i}+q\right]\times \left[x_{n}-p,x_{n}\right]$ and $T_{2}=\left[q,2q\right]\times {⨂}_{j=2}^{n-1}\left[z_{i},z_{i}+q\right]\times \left[x_{n}+p,x_{n}+p+q\right]$ where $y_{i},z_{i}\in \left(x_{i}-q,x_{i}+\varepsilon +q\right)$ for all i∈[2,n−1]. Now $E:=\left[q+p,2q\right]\times {⨂}_{j=2}^{n-1}\left[x_{i},x_{i}+\varepsilon \right]\times \left[x_{n},x_{n}+p\right]$ cannot be filled by any cubes, because it touches a small cube and it touches *T*_1_ and *T*_2_ in opposite full sides of distance *p* (see Figure 3).

**FIGURE 4:**
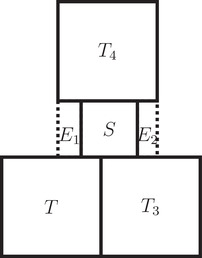
The 2‐dimensional setup of the second case in the proof of Lemma 4

Now assume that *S* does not touch *T* in a full side. Without loss of generality let $x_{n}>q-p$ and let $x_{i}\in \left[0,q\right)$ for all i∈[2,n−1]. Let *T*_3_ be the big cube properly touching *S* in $\left\{q\right\}\times {⨂}_{j=2}^{n-1}\left[x_{i},x_{i}+\varepsilon \right]\times \left[q,q+\varepsilon \right]$ and let *T*_4_ be the big cube properly touching *S* in $\left\{q+p\right\}\times {⨂}_{j=2}^{n}\left[x_{i},x_{i}+\varepsilon \right]$ for a ε>0. We write $T_{4}=\left[q+p,2q+p\right]\times {⨂}_{j=2}^{n}\left[w_{i},w_{i}+q\right]$ with $w_{i}\in \left[x_{i}-q+\varepsilon ,x_{i}\right]$ for all i∈[2,n−1]. If $w_{n}<x_{n}$, then $E_{1}:=\left[q,q+p\right]\times {⨂}_{j=2}^{n-1}\left[x_{i},x_{i}+\varepsilon \right]\times \left[w_{n},x_{n}\right]$ cannot be filled by any cubes, because it touches a small cube and it touches *T* and *T*_4_ in opposite full sides of distance *p*. Otherwise $E_{2}:=\left[q,q+p\right]\times {⨂}_{j=2}^{n-1}\left[x_{i},x_{i}+\varepsilon \right]\times \left[q,x_{n}+p\right]$ cannot be filled by any cubes, because it touches a small cube and it touches *T*_3_ and *T*_4_ in opposite full sides of distance *p* (see Figure 4).□

Lemma 5 Let *S* be a small cube with center *s* and T a big cube with center s+t that properly touches *S*. Then there is a big cube with center s−t in the tiling.

Without loss of generality let $S:={\left[0,p\right]}^{n}$ and $T:=\left[-q,0\right]\times {\left[0,q\right]}^{n-1}$ and let $T^{\prime }$ be the big cube properly touching *S* in the hyperplane $\left\{p\right\}\times R^{n-1}$ and write $T^{\prime }=\left[p,p+q\right]\times {⨂}_{i=2}^{n}I_{i}$ with $I_{i}\in \left\{\left[0,q\right],\left[p-q,p\right]\right\}$. Assume to the contrary that $T^{\prime }\neq \left[p,p+q\right]\times {\left[p-q,p\right]}^{n-1}$ and let l∈[2,n] such that $I_{l}=\left[0,q\right]$. Then $E:={\left[0,p\right]}^{l-1}\times \left[p,q\right]\times {\left[0,p\right]}^{n-l}$ touches *T* and $T^{\prime }$ with a full side in parallel hyperplanes with distance *p*, so it can only be filled with small cubes. This contradicts Lemma 3 because *E* touches *S*.□

Now assume $T:={\left[0,q\right]}^{n}$ and $S:={\left[0,p\right]}^{n-1}\times \left[q,q+p\right]$ are two cubes in the tiling. Let $T_{i}$ be the cube properly touching *S* in the hyperplane $R^{i-1}\times \left\{p\right\}\times R^{n-i}$. Then $T_{i}={⨂}_{j=1}^{i-1}I_{i}^{\left(j\right)}\times \left[p,p+q\right]\times {⨂}_{j=i+1}^{n-1}I_{i}^{\left(j\right)}\times \left[q,2q\right]$ with $I_{i}^{\left(j\right)}\in \left\{\left[0,q\right],\left[p-q,p\right]\right\}$. Since $T_{i}$ and $T_{j}$ do not properly intersect, we have $I_{i}^{\left(j\right)}=\left[p-q,p\right]$ or $I_{j}^{\left(i\right)}=\left[p-q,p\right]$. From Lemma 5, we know that $T_{i}^{\prime }:={⨂}_{j=1}^{i-1}J_{i}^{\left(j\right)}\times \left[-q,0\right]\times {⨂}_{j=i+1}^{n-1}J_{i}^{\left(j\right)}\times \left[p,p+q\right]$ with

$$J_{i}^{\left(j\right)}=\left\{\begin{array}{cc} \left[0,q\right] & I_{i}^{\left(j\right)}=\left[p-q,p\right]\\ \left[p-q,p\right] & I_{i}^{\left(j\right)}=\left[0,q\right] \end{array}\right.$$

is also a cube in the tiling. Since $T_{i}^{\prime }$ and $T_{j}$ cannot properly intersect, $J_{i}^{\left(j\right)}=\left[p-q,p\right]$ or $I_{j}^{\left(i\right)}=\left[0,q\right]$ and consequently $\left\{I_{i}^{\left(j\right)},I_{j}^{\left(i\right)}\right\}=\left\{\left[0,q\right],\left[p-q,p\right]\right\}$.

Lemma 6 There exists exactly one i∈[1,n−1] such that $T_{i}={⨂}_{j=1}^{i-1}\left[0,q\right]\times \left[p,p+q\right]\times {⨂}_{j=i+1}^{n-1}\left[0,q\right]\times \left[q,2q\right]$.

**FIGURE 5:**
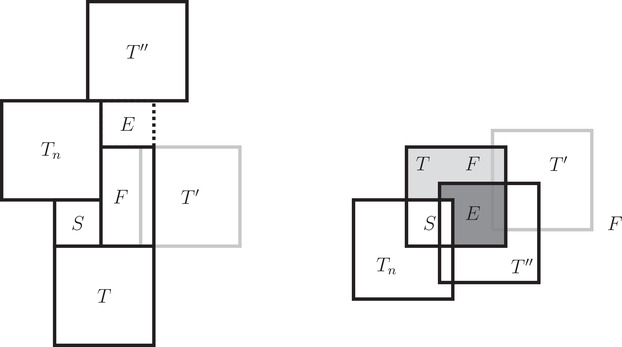
The 3‐dimensional setup in the proof of Lemma 6 seen in cross‐section (left) and from above (right)

From Lemma 5, we know that $T_{n}:={\left[p-q,p\right]}^{n-1}\times \left[q+p,2q+p\right]$ is also a cube in the tiling. Let $T^{\prime }$ be such that $T^{\prime }$ touches *T* in ${\left[q-\varepsilon ,q\right]}^{n-1}\times \left\{q\right\}$ then $T^{\prime }={⨂}_{j=1}^{n-1}\left[x_{i},x_{i}+q\right]\times \left[q,2q\right]$ with $x_{j}\in \left[0,q\right)$. Let $T^{\prime \prime }$ be such that $T^{\prime \prime }∼T_{n}=T^{\prime }∼T$, then $T^{\prime \prime }={⨂}_{j=1}^{n-1}\left[p-q+x_{i},p+x_{i}\right]\times \left[2q+p,3q+p\right]$. Since *S* is the unique small cube properly touching *T* in $R^{n-1}\times \left\{q\right\}$, $F:=({\left[0,q\right]}^{n-1}\setminus {\left[0,p\right]}^{n-1})\times \left[q,2q\right]$ is filled by big cubes properly touching *T*. Now consider $E:=\left({⨂}_{j=1}^{n-1}\left[p-q+x_{i},p+x_{i}\right]\cap \left({\left[0,q\right]}^{n-1}\setminus {\left[0,p\right]}^{n-1}\right)\right)\times \left[2q,2q+p\right]$. *E* is touching *F* and $T^{\prime \prime }$ in full opposite sides of distance *p* so it can only by filled by small cubes and since small cubes cannot touch, by at most one small cube (see Figure 5).

There is an i∈[1,n−1] such that $x_{i}⩾p$ otherwise $T^{\prime }$ and *P* would properly intersect so without loss of generality assume $x_{1}⩾p$

Claim 1 $x_{i}=0$ for all i∈[2,n−1].

Proof of Claim 1 Assume without loss of generality $x_{2}>0$ for k∈[2,n−1]. Then $x_{1}:={\left(p-3\varepsilon ,p+\varepsilon ,p-\varepsilon ,\text{…},p-\varepsilon ,2q+\varepsilon \right)}^{T}$ and $x_{2}:={\left(p+\varepsilon ,p-3\varepsilon ,p-\varepsilon ,\text{…},p-\varepsilon ,2q+\varepsilon \right)}^{T}$ are inner points of *E* but their midpoint $x_{m}:=\left(x_{1}+x_{2}\right)/2={\left(p-\varepsilon ,\text{…},p-\varepsilon ,2q+\varepsilon \right)}^{T}$ is an inner point of $T_{n}$ so *E* cannot be filled by a single convex set.□

Claim 2 It holds that $x_{1}=p$.

Proof of Claim 2 Assume $x_{1}>p$. Then $E^{\prime }:=\left[p,x_{1}\right]\times {\left[0,p\right]}^{n-2}\times \left[q,p+q\right]$ is touching $T^{\prime }$ and *S* in full opposite sides of distance at most q−p so $E^{\prime }$ cannot be filled.□

Therefore $T^{\prime }=\left[p,p+q\right]\times {\left[0,q\right]}^{n-2}\times \left[q,2q\right]$ and since two different cubes of the form ${⨂}_{j=1}^{i-1}\left[0,q\right]\times \left[p,p+q\right]\times {⨂}_{j=i+1}^{n-1}\left[0,q\right]\times \left[q,2q\right]$ would intersect, $T^{\prime }$ is the unique cube of this form.□

**FIGURE 6:**
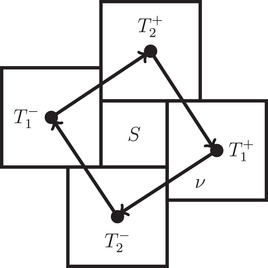
The 2‐dimensional setup for Lemma 7

Now again let *S* be a small cube in the tiling and without loss of generality let $S:={\left[-p/2,p/2\right]}^{n}$. Let $T:=\{T_{1}^{-},\text{…},T_{n}^{-},T_{1}^{+},\text{…},T_{n}^{+}\}$ be the set of cubes properly touching *S* such that *S* touches $T_{i}^{-}$ in $R^{i-1}\times \left\{-p/2\right\}\times R^{n-i}$ and $T_{i}^{+}$ in $R^{i-1}\times \left\{p/2\right\}\times R^{n-i}$ for all i∈[1,n] and $C:=\{c_{1}^{-},\text{…},c_{n}^{-},c_{1}^{+},\text{…},c_{n}^{+}\}$ their corresponding center point. We can now define a function ν:T→T that assigns every cube T∈T the cube $T^{\prime }$ such that $\left(S,T,T^{\prime }\right)$ are in the same relation as $\left(S,T,T_{i}\right)$ from Lemma 6 with the uniquely determined *i*. We also use ν as a function ν:C→C with the obvious meaning. Consequently, for all i∈[1,n] there exists j∈[1,n]∖{i} such that $\nu \left(v_{i}^{-}\right)-v_{i}^{-}=qe_{i}\pm pe_{j}$ and for symmetry reasons $\nu \left(v_{i}^{+}\right)-v_{i}^{+}=-qe_{i}\mp pe_{j}$. Now let G:=(V,E) be the directed graph with vertex set V:=T and edge set E:={(T,ν(T))|T∈T} (see Figure 6).

Lemma 7 *G* is a cycle of length *n* and $\nu^{n}\left(T_{i}^{\pm }\right)=T_{i}^{\mp }$.

Every vertex in *G* has out‐degree one so *G* contains a cycle. Let $C:=({\bar{T}}_{1},\text{…},{\bar{T}}_{k})$ be a cycle in *G* with center points $\left({\bar{c}}_{1},\text{…},{\bar{c}}_{k}\right)$, ${\bar{T}}_{1}={\bar{T}}_{k}$ and 3⩽k⩽2n+1. For i∈[1,k−1], let $j_{i}\in \left[1,n\right]$ such that ${\bar{T}}_{i}\in \left\{T_{j_{i}}^{-},T_{j_{i}}^{+}\right\}$. Then ${\bar{c}}_{i}$ can be written as

$${\bar{c}}_{i}=x_{i}\frac{q+p}{2}e_{j_{i}}+x_{i+1}\frac{q-p}{2}e_{j_{i+1}}+\sum_{l\in S_{i}^{-}}\frac{p-q}{2}e_{l}+\sum_{l\in S_{i}^{+}}\frac{q-p}{2}e_{l},$$

where $\left\{j_{i},j_{i+1}\right\}⊎S_{i}^{-}⊎S_{i}^{+}=\left[1,n\right]$ and $x_{i},x_{i+1}\in \left\{-1,1\right\}$ and

$${\bar{c}}_{i+1}=-x_{i}\frac{q-p}{2}e_{j_{i}}+x_{i+1}\frac{q+p}{2}e_{j_{i+1}}+\sum_{l\in S_{i}^{-}}\frac{p-q}{2}e_{l}+\sum_{l\in S_{i}^{+}}\frac{q-p}{2}e_{l}.$$

Let $v_{i}:={\bar{c}}_{i+1}-{\bar{c}}_{i}=-x_{i}qe_{j_{i}}+x_{i+1}pe_{j_{i+1}}$, then $\sum_{i=1}^{k-1}v_{i}=0$ because $c_{k}=c_{1}$.

$$0=\sum_{i=1}^{k-1}v_{i}=\sum_{i=1}^{k-1}-x_{i}qe_{j_{i}}+x_{i+1}pe_{j_{i+1}}=\sum_{i=1}^{k-1}x_{i}\left(p-q\right)e_{j_{i}}.$$

Therefore $T_{j}^{-}\in C\Leftrightarrow T_{j}^{+}\in C$ for all j∈[1,n] and *C* is an even cycle. Without loss of generality let ${\bar{T}}_{1}=T_{1}^{-}$, then for symmetry reasons ${\bar{T}}_{k/2+1}=T_{1}^{+}$ and

$$\left(p+q\right)e_{1}+\sum_{l=2}^{n}\pm \left(q-p\right)e_{l}=v_{k/2+1}-v_{1}=\sum_{i=1}^{k/2}v_{i}=\sum_{i=1}^{k/2}-x_{i}qe_{j_{i}}+x_{i+1}pe_{j_{i+1}},$$

where $e_{j_{1}}=e_{j_{k/2+1}}=e_{1}$ and $\left\{e_{j_{1}},\text{…},e_{j_{k/2}}\right\}$ are pairwise different. Consequently, k/2=n which concludes the proof.□

Now consider the sets of vectors $\left\{b_{1},b_{2},\text{…},b_{n}\right\}\subseteq BZ^{n}$ where

$$b_{i}={\bar{c}}_{i+1}-{\bar{c}}_{i}=\left\{\begin{array}{cc} pe_{j}+qe_{l} & {\bar{T}}_{i+1}=T_{j}^{+}\wedge {\bar{T}}_{i}=T_{l}^{-}\\ -pe_{j}+qe_{l} & {\bar{T}}_{i+1}=T_{j}^{-}\wedge {\bar{T}}_{i}=T_{l}^{-}\\ pe_{j}-qe_{l} & {\bar{T}}_{i+1}=T_{j}^{-}\wedge {\bar{T}}_{i}=T_{l}^{+}\\ -pe_{j}-qe_{l} & {\bar{T}}_{i+1}=T_{j}^{-}\wedge {\bar{T}}_{i}=T_{l}^{+}. \end{array}\right.$$

Let $C:=(b_{1},b_{2},\text{…},b_{n})$, then SC=A where $S={\left(s_{i,j}\right)}_{i,j\in [1,n]}\in B_{n}^{\prime }$ and

$$s_{i,j}=\left\{\begin{array}{cc} 1 & {\bar{T}}_{i}=T_{j}^{-}\\ -1 & {\bar{T}}_{i}=T_{j}^{+}\\ 0 & else. \end{array}\right.$$

Now $\det\left(C\right)=\det\left(A\right)=\det\left(B\right)=p^{n}+q^{n}$ and therefore $CZ^{n}=BZ^{n}$ and *A* and *B* describe the same tiling up to symmetries.

## JOURNAL INFORMATION

*Mathematika* is owned by University College London and published by the London Mathematical Society. All surplus income from the publication of *Mathematika* is returned to mathematicians and mathematics research via the Society's research grants, conference grants, prizes, initiatives for early career researchers and the promotion of mathematics.
